# Characterization of Hypolipidemic Phenol Analogues from Fermented Tea by *Eurotium cristatum*

**DOI:** 10.3390/foods12010049

**Published:** 2022-12-22

**Authors:** Fuhang Song, Wei Dai, Honghua Li, Xinwan Zhang, Xiuli Xu, Linlin Ma, Long Wang

**Affiliations:** 1 School of Light Industry, Beijing Technology and Business University, Beijing 100048, China; 2 School of Ocean Sciences, China University of Geosciences, Beijing 100083, China; 3 Griffith Institute for Drug Discovery, School of Environment and Science, Griffith University, Brisbane, QLD 4111, Australia; 4 Key Laboratory of Mycology, Institute of Microbiology, Chinese Academy of Sciences, Beijing 100101, China

**Keywords:** Fuzhuan brick tea, hydroxybenzaldehyde derivatives, *Eurotium cristatum*, hypolipidemic, fatty liver, C57BL/6N mice

## Abstract

Fuzhuan brick tea (FBT), a type of black tea, is a traditional beverage in China, especially popular among frontier ethnic groups. FBT is well-known for its health benefits, such as hypoglycemic, anti-hypertensive, anti-inflammatory, diuretic, and detoxification effects. Nevertheless, the underlying mechanisms on the molecular level are still elusive and the key compounds responsible for the health benefits are unidentified. Previous studies have mainly focused on functional studies of the water extract. However, FBT is typically cooked with butter or milk. Therefore, we hypothesized that some lipophilic components in FBT, which can be absorbed through the co-consumption of butter or milk, may play an important role in the health benefits. The present study aimed to investigate whether the liposoluble extract of FBT alleviates symptoms related to metabolic diseases and to identify the active compounds involved. By comparing the high-performance liquid chromatography (HPLC) profiles of water, milk and hexane extract, some low polarity peaks were observed in the milk and hexane extracts. Furthermore, the hexane extract treatment alleviated body weight gain, serum total cholesterol and triglyceride levels, and inhibited the accumulation of hepatic fat granules in a high-fat diet (HFD)-induced C57BL/6N mouse model. In order to identify the key functional lipophilic compounds in FBT, the hexane extract of FBT was subjected to chemical characterization. Four phenol analogs were characterized, namely, isodihydroauroglaucin (1), dihydroauroglaucin (2), tetrahydroauroglaucin (3), and flavoglaucin (4). Compounds **1** and **4** reduced the levels of total cholesterol and triglyceride in vivo. Both compounds also inhibited the high-fat diet-induced body weight gain and accumulation of fat granules in the liver of C57BL/6N mice. Isodihydroauroglaucin and flavoglaucin have therefore been identified as bioactive ingredients that contribute to the health benefits of FBT.

## 1. Introduction

Metabolic syndrome (MetS) is characterized by a set of abnormalities in metabolism, resulting in a clustering of at least three of the five insulin resistance-associated medical diseases, including high blood pressure, abdominal obesity, type 2 diabetes, high serum triglycerides, and low serum high-density lipoprotein (HDL) [1,2,3]. It was estimated that the global prevalence of MetS in 2020 was 2.8% for children and 4.8% for adolescents, meaning 25.8 million children and 355 million adolescents are living with MetS [3]. The prevalence of MetS was over 20% among adults in both developed and developing countries [4,5,6]. The dramatic increase in the prevalence of MetS is thought to be driven by modern dietary habits, such as more consumption of fast food, highly processed foods, and high-fat diets [7,8]. There are many drugs available to treat these medical conditions, but the significant side effects due to the long-term use of these drugs pose a high threat to public health [9,10,11]. Therefore, new drugs with enhanced efficacy and decreased side effects [12], and/or dietary strategies [13] are vital approaches to reduce the impact of MetS.

Functional foods and beverages represent the most attractive dietary strategies for controlling metabolic syndrome in food and nutrition sciences. Animal studies have demonstrated that polyphenol-rich foods can improve dyslipidemia and body fat distribution by changing microbial metabolites [14]. Consuming functional foods has been proven to be an efficient and convenient method to improve health and decrease the use of medicines [15,16]. Today, functional foods and beverages are rapidly growing in the market because they provide health benefits beyond the provision of essential nutrients. Many functional foods claim that they are derived from traditional food components, but the functional activities of these products must be confirmed by scientific evidence [17]. Therefore, in vitro and in vivo studies should be carried out during the development of new products [18,19].

Fuzhuan brick tea (FBT), a type of black tea, is a kind of fermented tea that has been reported to have health benefits because of the fermentation process involving, predominantly, the microorganism *Eurotium cristatum*. FBT, also known as ‘Jingyang Zhuan’, is believed to have originated from Jingyang, Shaanxi Province in China, with a long history dating back to the 16th century [20,21]. FBT was mainly consumed in the border regions of Southwestern and Northwestern China by ethnic groups, such as Uygurs, Tibetans, and Mongolians [22]. Ethnic groups used to consume a lot of meat and milk and minimal vegetables and fruits, which is considered an unhealthy diet from the point of view of modern nutrition. Consumption of FBT has been playing an important role in keeping these ethnic groups healthy [23]. Due to its unique flavor and health-promoting functions, FBT has attracted lots of investigations on the microorganisms [24,25], chemical constituents [21,26,27,28,29,30], and functions of its water extract [31,32,33,34]. However, limited studies have been performed to identify the key functional compounds from FBT. 

FBT is usually consumed together with milk and butter, suggesting that the health benefits of FBT might come from its lipophilic components. This study aimed to evaluate the health beneficial effects of the liposoluble extract of FBT on MetS in a high-fat diet-induced mouse model. Furthermore, low-polarity compounds’ characterization from the hexane extract of FBT and their functional evaluation in a mouse model were also investigated.

## 2. Materials and Methods

### 2.1. Preparation of FBT, Extraction, and Compounds Isolation

FBT (2 kg, donated by Bio-tea Co. Ltd. of Shaanxi Biotech Group, Xi’an, Shaanxi Province, China) was extracted three times with 10 L EtOH, and the organic solvent was evaporated in vacuo with a rotary evaporator at 45 °C to yield a brown crude extract. The crude extract was resuspended in 500 mL distilled water and extracted with 500 mL hexane (three times). Hexane was then evaporated in vacuo at 45 °C, yielding a dark liposoluble extract (26.2 g). The hexane extract was further fractionated by a vacuum liquid silica gel chromatography (80 × 80 mm column, Silica gel 60 H for thin-layer chromatography) using a stepwise gradient of 100% hexane to 10% hexane in CH_2_Cl_2_ to afford 9 fractions. The fractions were subjected to a Sephadex LH-20 column using an isocratic elution of CH_2_Cl_2_:MeOH (2:1) to yield subfractions, which were further fractionated by reversed-phase HPLC (Agilent Eclipse XDB-C8, 250 × 9.4 mm, 5 μm column, 3.0 mL/min, elution with 40% to 80% acetonitrile/H_2_O over 20 min, then keeping 80% acetonitrile/H_2_O for 15 min) to yield compounds **1**–**4**.

Isodihydroauroglaucin (1): Light yellow powder; ^1^H and ^13^C NMR data, Table 1; HRESIMS *m/z* 323.1621 [M + Na]^+^ (calcd for C_19_H_24_O_3_Na, 323.1628).

Dihydroauroglaucin (2): Light yellow powder; ^1^H and ^13^C NMR data, Table 1; HRESIMS *m/z* 301.1798 [M + H]^+^ (calcd for C_19_H_25_O_3_, 301.1798).

Tetrahydroauroglaucin (3): Light yellow powder; ^1^H and ^13^C NMR data, Table 1; HRESIMS *m/z* 325.1781 [M + Na]^+^ (calcd for C_19_H_26_O_3_Na, 325.1774).

Flavoglaucin (4): Light yellow powder; ^1^H and ^13^C NMR data, Table 1; HRESIMS *m/z* 327.1929 [M + Na]^+^ (calcd for C_19_H_28_O_3_Na, 327.1931).

### 2.2. Structure Determination of Purified FBT Compounds

The structures of purified FBT compounds were determined based on the Nuclear Magnetic Resonance (NMR) analysis and high-resolution mass spectrum data. Compounds **1**–**4** (10 mg each) were dissolved in deuterated chloroform or pyridine and put into 5 mm NMR sample tubes, respectively. NMR spectra were obtained on a Bruker Avance 500 spectrometer with residual solvent peaks as references (Pyridine-*d*_5_: *δ*_H_ 7.22, *δ*_C_ 123.9; CDCl_3_: *δ*_H_ 7.26, *δ*_C_ 77.16). Compounds **1**–**4** in methanol (0.1 mg/mL) were subjected to high-resolution electrospray ionization mass spectrometry (ESI-MS) analysis with an Accurate-Mass-Q-TOF LC/MS 6520 instrument (Santa Clara, CA, USA) in the positive or negative ion mode.

### 2.3. DPPH Radical Scavenging Assay

The antioxidant activity of purified compounds from FBT was tested using DPPH radical scavenging assay using the previously reported method [35]. Briefly, 1,1-Diphenyl-2-picryl-hydrazyl (DPPH) and trolox (from Shanghai Acmec Biochemical Co., Ltd., Shanghai, China) were used as negative and positive controls, respectively. The tested samples were dissolved in DMSO at pre-designed concentrations and incubated with an equal volume (100 µL) of DPPH (40 µg/mL in ethanol) at 30 °C for 30 min in dark. The absorbance was performed at 517 nm using ST-360 spectrophotometer (Shanghai Kehua Laboratory System Co., Ltd., Shanghai, China).

### 2.4. Experimental Design for Animal Studies

Male (20–25 g, eight-week-old) C57BL/6N mice were purchased from Beijing Vital River Laboratory Animal Technology Co., Ltd. (Beijing, China). All procedures were carried out in accordance with the Care and Use of Laboratory Animals of People’s Republic of China. Mice were kept in an animal room at 23 °C with a 12-h light/dark cycle and acclimated for 7 days before the studies were performed. The obesity group (40 mice) was fed with a high fat diet (HFD, 0.2% cholesterol, 15% lard oil) and the control group (10 mice) was fed with a normal diet (ND). After 8 weeks, 40 mice fed with the HFD were divided into four sub-groups (non-treated positive control group (HFD), hexane extract treated group (HET), compound **1** treated group (CT1), and compound **4** treated group (CT4)). The mice in the ND and HFD groups were administered intragastrically with 0.5% carboxymethylcellulose sodium once daily for eight weeks. The mice in HET, CT1, and CT4 groups were administered intragastrically based on body weight with 500 mg/kg hexane extract and 5 mg/kg of compounds **1** and **4** once daily, respectively. After the last administration, all the mice fasted for 12 h before further analysis [24].

### 2.5. Biochemical Analysis

Blood was collected from the orbit of mice after being fasted for 12 h. The serum was separated from blood cells by centrifugation at 3000× *g* rpm for 5 min. Biochemical analysis of the total cholesterol (TC) and triglyceride (TG) in the serum was performed using a Triglyceride Assay Kit and Total Cholesterol Assay Kit (Nanjing Jiancheng Bioengineering Institute, Nanjing, China) following the manufacturer’s instructions [33].

### 2.6. Preparation for Liver Hematoxylin-Eosin Staining

The liver was collected after anesthesia by isoflurane, then washed with physiological saline and fixed in 4% formaldehyde solution for 72 h. Then, the fixed liver was embedded in paraffin and prepared for sliding with an HM 325 Rotary Microtome (Thermo Scientific, Waltham, MA, USA) for fat granules observation. The images of sections were captured using an OLYMPUS BX43 Light Microscope (OLYMPUS, Japan) [33].

### 2.7. Statistical Analysis

All experimental statistical analyses were performed using GraphPad Prism 8.0.2 (GraphPad Software, San Diego, CA, USA). At least three independent repeats were carried out for each experiment and data are reported as mean ± standard deviation (SD). Statistical significance of differences between groups was determined using unpaired Student’s *t*-test or ordinary one-way ANOVA. Statistical significance is denoted by * for *p* ≤ 0.05, ** for *p* ≤ 0.01, and *** for *p* ≤ 0.001.

## 3. Results

### 3.1. HPLC Analysis of the Water, Milk, and Hexane Extracts of FBT

**Figure 1:**
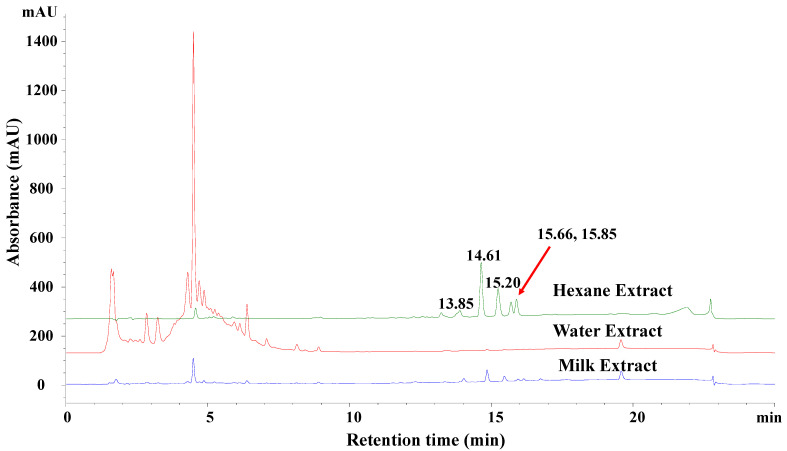
HPLC profiles of water (red), milk (blue), and hexane (green) extracts of FBT. The HPLC analysis was performed on a reversed-phase C8 column (XDB-C8, 4.6 × 150 mm, 5 μm, Agilent), eluting with 10–100% MeCN/H_2_O in 15 min, then keeping 100% MeCN for 5 min, and equating with 10% MeCN/H_2_O for 5 min. Absorbance was monitored at 254 nm.

FBT is usually cooked with butter or milk. During the cooking process, some of the less polar constituents in FBT, which may have health benefits, could be extracted into the fat in milk or butter. Therefore, the reversed-phase HPLC profiles of water, milk, and hexane extracts were analyzed and compared. As shown in Figure 1, some less polar HPLC peaks with retention times of 13.85 min, 14.61 min, 15.20 min, 15.66 min, and 15.85 min were observed in the hexane extract, and to a lesser extent, in the milk extract, but not in the water extract. The results suggested that these low-polarity components might have been missed in previous studies focusing exclusively on the FBT water extracts.

### 3.2. Compounds Isolation and Structure Elucidation

**Figure 2:**
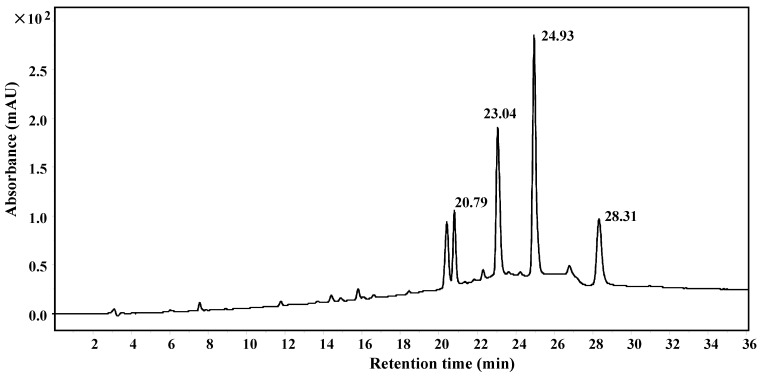
HPLC purification of the FBT components soluble in hexane. The hexane-specific peaks in the FBT crude extract were further purified using an Agilent 1200 HPLC system (Agilent Eclipse XDB-C8, 250 × 9.4 mm, 5 μm column) using a gradient of acetonitrile from 40% to 80% acetonitrile/H_2_O over 20 min, followed by 80% acetonitrile/H_2_O for 15 min at a flow rate of 3.0 mL/min. Absorbance was monitored at 254 nm.

The lipophilic components presented in the milk/hexane extracts were more enriched in the hexane extract (Figure 1). Therefore, the hexane extract was further fractionated with reduced pressure silica gel chromatography, Sephadex LH-20 chromatography, and HPLC in order to isolate each compound contained in the peaks with retention times of 14.61 min, 15.20 min, 15.66 min, and 15.85 min (as in Figure 1) for structural studies. As shown in Figure 2, fractions containing the above peaks were further purified using reversed-phase HPLC with an Eclipse XDB-C8 column to yield compounds **1**–**4**, with retention times of 20.79, 23.04, 24.93, 28.31 min, respectively.

**Figure 3:**
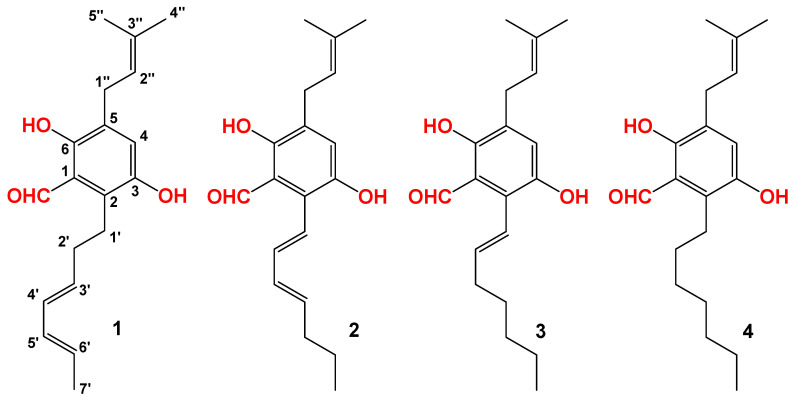
The structures of isolated compounds from the hexane-extracted fractions of FBT.

Compound **1** was isolated as a light yellow powder. The molecular formula of **1** was determined to be C_19_H_24_O_3_ based on the HRESIMS spectrum (*m/z* [M + Na]^+^ 323.1621, calcd for C_19_H_24_O_3_Na^+^, 323.1618), accounting for seven degrees of unsaturation (Figure S1). The ^1^H and ^13^C NMR spectra of **1** (Table 1, Figures S2 and S3) displayed signals for 5-substituted (including two hydroxyl) benzene rings, one isoprenoid moiety, and one heptadiene moiety. All of these data were similar to those of isodihydroauroglaucin (IDAG). The structure of isodihydroauroglaucin was assigned as *trans* conformation for all of the olefinic protons [36]. By analyzing the coupling constants of H-3′ at *δ*_H_ 5.78 (dt, *J* = 14.0, 8.0 Hz), H-4′ at *δ*_H_ 6.16 (dd, *J* = 14.0, 10.0 Hz), H-5′ at *δ*_H_ 6.08 (dd, *J* = 14.0, 10.0 Hz), H-6′ at *δ*_H_ 5.56 (dq, *J* = 14.0, 7.0 Hz), the geometric configurations between of H-3′ and H-4′, H-4′ and H-5′, H-5′ and H-6′ were *trans*, *cis*, and *trans*, respectively. Therefore, the geometric configurations were revised, as shown in Figure 3.

Compound **2** was also isolated as a light yellow powder. The molecular formula of compound **2** was determined to be C_19_H_24_O_3_ based on the HRESIMS spectrum (*m/z* [M + H]^+^ 301.1798, calcd for C_19_H_25_O_3_, 301.1798), accounting for seven degrees of unsaturation (Figure S4). The ^1^H and ^13^C NMR spectra of **1** (Table 1, Figures S5 and S6) displayed similar signals as those of dihydroauroglaucin (DAG). The structure of dihydroauroglaucin was assigned with *trans* conformation for all of the olefinic protons [30]. By analyzing the coupling constants of H-1′ at *δ*_H_ 6.56 (d, *J* = 16.0 Hz), H-2′ at *δ*_H_ 6.44 (dd, *J* = 16.0, 10.0 Hz), H-3′ at *δ*_H_ 6.27 (dd, *J* = 14.5, 10.0 Hz), and H-4′ at *δ*_H_ 5.89 (dt, *J* = 14.5, 7.5 Hz), the geometric configurations between of H-1′ and H-2′, H-2′ and H-3′, and H-3′ and H-4′ were *trans*, *cis*, and *trans*, respectively. Therefore, the geometric configurations of dihydroauroglaucin were revised, as shown in Figure 3.

Compounds **3** and **4** were identified as tetrahydroauroglaucin and flavoglaucin, respectively, by comparison of molecular formula and NMR data (Table 1, Figures S7–S12) with reported data [35]. 

### 3.3. Antioxidant Activity of Compounds

**Figure 4:**
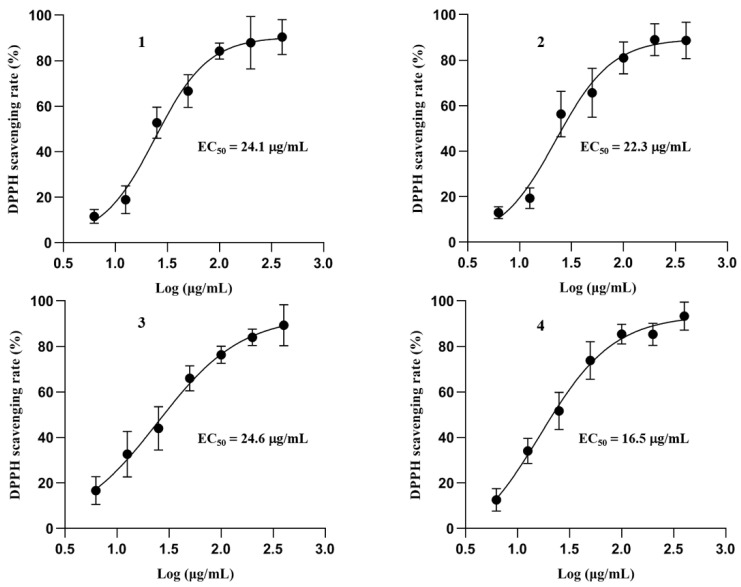
Antiradical activities of isolated phenol analogues **1**–**4**. Dose-dependent antioxidant activities of compounds **1**–**4** were tested with DPPH radical scavenging assay. Data are presented as mean ± SD (*n* = 3).

Phenol compounds are considered to display antioxidant activity because of the hydroxyls in their structures [37,38]. Previous studies have reported that flavoglaucin analogs from *E. herbariorum* exhibited radical scavenging activity [35]. In order to evaluate the antioxidant potential of the purified compounds and set a foundation for further in vivo study, all of the compounds were tested with DPPH radical scavenging assay by using trolox as positive control (with EC_50_ value of 29.32 μg/mL). These compounds displayed potent antioxidant activity by scavenging the DPPH radical effectively, with EC_50_ ranging from 16.5 μg/mL (Compound **4**) to 24.6 μg/mL (Compound **3**) (Figure 4).

### 3.4. Hexane Extract, Compounds ***1*** and ***4*** Alleviated HFD-Induced Metabolic Syndrome in C57BL/6N Mice

**Figure 5:**
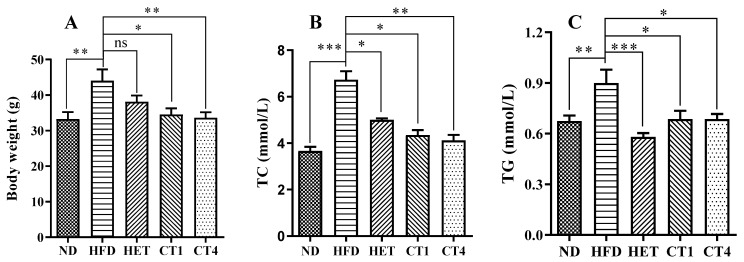
The effect of FBT hexane extract and isolated compounds on body weight and dyslipidemia. (**A**): body weight; (**B**): serum total cholesterol; (**C**): serum triglyceride. ND: normal diet treated group; HFD: high-fat diet; HET: high-fat diet and hexane extract treated group; CT1: high-fat diet and compound 1 treated group; CT4: high-fat diet and compound **4** treated group. Data are expressed as the mean SEM (*n* = 10). Statistical significance is denoted by * for *p* ≤ 0.05, ** for *p* ≤ 0.01, and *** for *p* ≤ 0.001.

In order to investigate the biological effects of hexane-solubilized components of FBT, and isolated compounds, total hexane extract (HET group), compound **1** (CT1 group) and compound **4** (CT4 group) were administered to high-fat diet (HFD)-induced metabolic syndrome model mice for eight weeks. Three key metabolic syndrome indicators, namely, body weight, plasma total cholesterol (TC), and triglyceride (TG) were quantified. As shown in Figure 5A, after 16 weeks (24-week-old mice) of HFD feeding, the body weight of the HFD group was significantly higher than that of the normal diet treated group (ND group). The HET group (500 mg/kg) lost weight compared to the HFD group, but the difference was not statistically significant. In contrast, treatments with compounds **1** and **4** (5 mg/kg, respectively) both significantly relieved the HFD-induced body weight gain (*p* < 0.05, Figure 5A). 

To investigate the effects of the FBT treatments on dyslipidemia in the HFD-fed mice, serum total cholesterol (TC) and triglyceride (TG) levels were evaluated. The serum TC level significantly increased from 3.66 ± 0.57 mmol/L in the ND group to 6.73 ± 1.16 mmol/L in the HFD group (*p* < 0.05, Figure 5B). Similarly, the serum TG levels were significantly elevated in the HFD group (0.90 ± 0.25 mM) compared to the ND control group (0.67 ± 0.11 mM) (*p* < 0.05, Figure 5B). Notably, after 8 weeks of treatment with HET, the TC and TG levels were decreased to 5.01 ± 0.20 mM and 0.58 ± 0.07 mM, respectively, both significantly different from the HFD positive control group (*p* < 0.05, Figure 5B,C). CT1 and CT4 were even more effective than HET in reducing the TC levels in the mice, bringing the serum TC levels to 4.12 ± 0.74 mM and 4.34 ± 0.66 mM, respectively (*p* < 0.05 compared to HFD group, Figure 5B). Both CT1 and CT4 treatments also led to a reduction of TG levels to 0.69 ± 0.16 mM and 0.69 ± 0.10 mM, respectively, significantly alleviating the high triglyceride levels caused by the high-fat diet (*p* < 0.05 compared to HFD group, Figure 5C). 

These findings strongly suggest that the low-polarity components of FBT, especially the phenol analogs of flavoglaucin, have significant health benefits in promoting lipid homeostasis and reducing body weight gain in a metabolic syndrome mouse model.

### 3.5. Haxane Extract of FBT, Compounds ***1*** and ***4*** Inhibit the Accumulation of Fat Granules in the Liver of HFD-Fed C57BL/6N Mice

**Figure 6:**
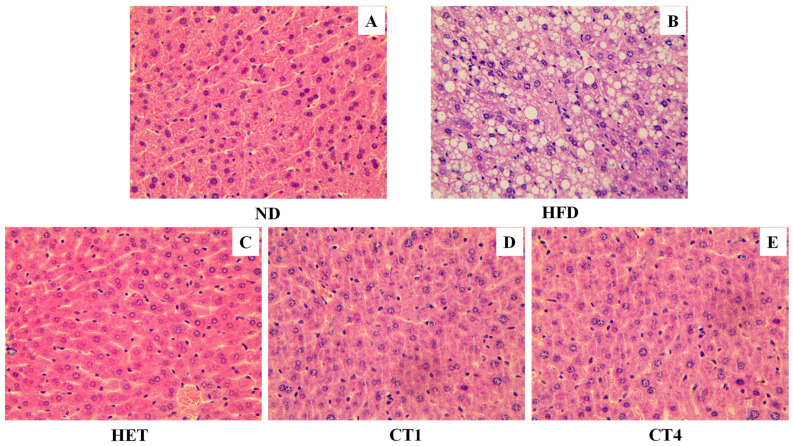
The effect of FBT hexane extract and isolated compounds on the accumulation of fat granules in the liver of C57BL/6N mice. (**A**) (ND): normal diet treated group; (**B**) (HFD): high-fat diet; (**C**) (HET): high-fat diet and hexane extract treated group; (**D**) (CT1): high-fat diet and compound 1 treated group; (**E**) (CT4): high-fat diet and compound 4 treated group.

The HFD-induced metabolic syndrome mouse model also features non-alcoholic fatty liver disease (NAFLD) due to dyslipidemia [39]. Histologically, the excessive fat build-up in the liver with NAFLD can be observed as hepatic fatty granules, which are composed of a core formed by triacylglycerol and sterol esters and a phospholipid monolayer surrounding the core. Inspired by the notable protective effects of HET, compound **1** and compound **4** on dyslipidemia, we further examined their effects on the formation of hepatic fatty granules. As shown in Figure 6, compared with control mice (ND group), a HFD induced a profound accumulation of lipid droplets in the liver of C57BL/6N mice (Figure 6B). Notably, after 8 weeks of treatment with HET and compounds **1** and **4**, the fat granules decreased significantly to a level comparable to the ND control group (Figure 6C–E vs. Figure 6A). These results revealed that the lipophilic compounds in FBT are highly effective in reducing the risk of high-fat diet-induced fatty liver. 

## 4. Discussion

Metabolic syndrome (MetS) includes a series of different clinical symptoms, such as high blood pressure, adiposity, type 2 diabetes, dyslipidemia, and non-alcoholic fatty liver disease (NAFLD), which were all caused by abnormal metabolism [40]. In China, the incidence of MetS increased from 8 to 10.6% in urban areas and 4.9 to 5.3% in rural areas from 1992 to 2002 [41]. From USA CDC data from 2017, about 30.2 million adults aged 18 years or older (12.2% of USA adults) had type 2 diabetes mellitus, and one third of US adults had metabolic syndrome [42]. Usually, drug treatment is the first choice for metabolic syndrome, but long-term medication may cause many side effects. For example, statins are commonly used to treat dyslipidemia, but these drugs could cause muscle ache and discomfort (myalgia), with an incidence of 1–10%, and even rhabdomyolysis, which is a severe medical condition that can be fatal or result in permanent disability [43]. Rosiglitazone, an effective drug for type 2 diabetes mellitus, can lead to adverse events of edema, anemia, and weight gain [44].

Many functional foods and beverages have been proven effective in preventing or alleviating metabolic syndrome by modulating the body’s homeostasis mechanisms [33,45]. Specific foods, such as whole grains, low-fat dairy products, cheese, yogurt, olive oil, total fiber, dietary magnesium, and flavonoids, have demonstrated beneficial effects on type-2 diabetes mellitus [46,47]. However, detailed mechanistic studies are urgently needed to strengthen the application of function foods in the control of MetS.

FBT, a traditional Chinese beverage popular in the border region of China, has been considered to benefit metabolic disorders. It has been reported that the water extract suppresses the expression of inflammatory cytokines [48], attenuates high-fat diet-induced obesity and associated metabolic disorders [49,50], inhibits fat deposition [31], and protects the liver [33]. However, FBT is typically cooked with milk and butter, suggesting that the lipophilic components extracted into the fat of milk and butter may also contribute to the health benefits of FBT. To the best of our knowledge, no research has focused on the biological functions of the less polar constituents of FBT. In this study, the effects of lipophilic components from FBT on MetS were evaluated in a high-fat diet-induced C57BL/6N mouse model. After eight weeks of treatment with HET, TC and TG levels in the blood were both significantly decreased (Figure 5). Furthermore, the fat granules in the liver of MetS mice were significantly reduced to a level comparable to that ND controls. These results strongly support the health-beneficial functions of the lipophilic components of FBT on MetS. The composition of FBT has a superior health benefit compared to other tea because of the fermentation process during the manufacture of FBT, which involves unique microorganisms, specifically *E. cristatum* [51]. Due to the *E. cristatum*-dominated fermentation process, the level of major tea catechins, epigallocatechin gallate (EGCG) and epicatechin gallate (ECG), remarkably dropped to about 1/3 in the final product of FBT and four new B-ring fission metabolites of catechins Fuzhuanins C–F were generated [29]. Recently, four new acylglycosides flavones (camelliquercetiside E, camelliquercetiside F, camellikaempferoside D, and camellikaempferoside E) isolated from Fuzhuan brick tea were found to have inhibitory effects on α-glucosidase and HMG-CoA reductase, which may contribute to their hypoglycemic and hypolipidemic effects [30]. Interestingly, eurocristatine from the spore of *E. cristatum* has recently been shown to have hypoglycemic properties [25]. However, the primary compounds with anti-metabolic disorders functions in FBT remain elusive. Traditionally, FBT is cooked with milk or butter, so some fat-soluble compounds may be extracted into the tea soup during the cooking process. By comparing the HPLC profiles of the water extract, milk extract, and hexane extract of FBT, some low-polarity HPLC peaks were identified specifically in the milk and hexane extracts, but not in the water extract (Figure 1A). Chemical investigation of these lipophilic FBT components led to the characterization of four phenol analogs, namely, isodihydroauroglaucin (1), dihydroauroglaucin (2), tetrahydroauroglaucin (3), and flavoglaucin (4). All of these compounds belong to benzaldehyde-derived phenols, which are biosynthesized through the polyketide pathway [21,52]. Isodihydroauroglaucin (1) was first identified from the fungus of *A. ruber* [36]. It was also isolated from fungi of *Eurotium herbariorum* [53], *E. cristatum* [54], and *A. niveoglaucus* [55]. Nevertheless, all the previous studies presented the structure as a *trans* conformation for four olefinic protons of H-3′ and H-4′, H-4′ and H-5′, and H-5′ and H-6′. By detailed analysis of coupling constants, we deduced a *cis* conformation between H-4′ and H-5′. Isodihydroauroglaucin displayed a series of bioactivities, including antioxidant activity [35,56], germicidal activity against gram-positive bacteria [57], and anticancer effects in a mouse skin tumor model [58]. Flavoglaucin (4) has been isolated from *A. echinulatus* [59] and *E. repens* [60]. It exhibits a broad spectrum of bioactivities, such as antioxidant activity [35,53], antiviral activity against the HSV-1 virus [61], and selective inhibition of lung cancer cells [62]. Eurocristatine, characterized from *E. cristatum* spores, showed a significant hepatoprotective effect on diabetic rats when administered with a dosage of 30 mg per kg body weight [25]. In the present study, isodihydroauroglaucin (1) and flavoglaucin (4) (5 mg/kg, respectively) decreased the HFD-induced body weight gain (*p* < 0.05, Figure 5A). The serum levels of TG and TC in the HFD-induced symbolic syndrome mouse model were also reduced after eight weeks of treatment (*p* < 0.05 compared to HFD group, Figure 5B). Furthermore, both compounds alleviated the accumulation of lipid granules in the liver of MetS mice (Figure 6). Therefore, this study deciphered the health benefits of the traditional cooking methods of FBT.

Oxidative stress, acting as one of the essential regulators in the biological process, is a well-known key element involved in the development of diabetes, obesity, cardiovascular diseases, aging, cancer, and neurodegenerative diseases [43,63]. As metabolic disorders develop, upregulated redox signaling pathways generate excessive reactive oxygen species in the inflammatory environment in vivo, leading to the dysregulation of inflammatory cytokines, chemokines, and growth factors. These pathological alterations then promote the development of insulin resistance, diabetes, and cardiovascular damage [64]. Moreover, long-term disturbance of antioxidant responses will induce the development of non-alcoholic liver disease [65]. FBT displayed superior antioxidant activity than other teas. Moreover, the antioxidant activities of FBT were closely related to the levels of epigallocatechin gallate, theabrownins, and total flavonoids [66]. Polyphenols and flavonoids isolated from Pu-erh tea showed in vitro antioxidant activities by scavenging DPPH and ABTS [67]. Using a DPPH radical scavenging assay, our study showed that the phenolic compounds purified from the hexane fraction of FBT displayed significant antioxidant activity with a potency comparable to the positive control, Trolox (Figure 4). Our results are consistent with previous antioxidant studies of these compounds [35] and revealed the remarkable health benefits of phenolic compounds in FBT. Detailed studies on the mechanism of action, toxicity, dosage, and administration methods will facilitate the application of these phenolic compounds in the future.

## 5. Conclusions

The health benefits of FBT, especially for the symptoms associated with metabolic syndrome, have been a belief for thousands of years. However, comprehensive scientific evidence to support this belief has been lacking. In this study, we identified and characterized four new compounds, namely isodihydroauroglaucin, dihydroauroglaucin, tetrahydroauroglaucin, and flavoglaucin, from the hexane extract of FBT. The antioxidant activity of these phenol analogs at the cellular level and their therapeutic effects on metabolic syndrome in animal models demonstrate a novel mechanism of action for the health benefits of FBT. These findings also strongly support the traditional consumption methods of FBT, i.e., cooking FBT with milk and butter, which maximizes the benefits of this popular functional beverage.

## Figures and Tables

**Table 1:** ^1^H and ^13^C NMR data for compounds **1**–**4**.

Position	1 (Pyridine-*d*_5_)		2 (CD_3_Cl)		3 (CD_3_Cl)		4 (Pyridine-*d*_5_)	
	δ_H,_ Mult (*J* in Hz)	δ_C_	δ_H,_ Mult (*J* in Hz)	δ_C_	δ_H,_ Mult (*J* in Hz)	δ_C_	δ_H,_ Mult (*J* in Hz)	δ_C_
1		118.9		117.2		117.3		118.8
2		155.2		155.4		155.2		155.2
3		128.7		130.6		130.5		128.4
4	7.36, s	126.6	7.00, s	125.3	7.02, s	125.2	7.38, s	126.6
5		148.8		145.2		144.9		148.7
6		129.2		124.2		124.1		130.4
7	10.57, s	197.4	10.09, s	196.4	10.09, s	196.4	10.94, s	197.4
1′	3.36, t (8.0)	24.8	6.56, d (16.0)	119.5	6.48, d (16.5)	120.2	3.25, t (8.0)	24.7
2′	2.60, dt (8.0, 8.0)	35.2	6.44, dd (16.0, 10.0)	140.1	5.99, dt (16.5, 7.0)	142.9	1.77, m	33.0
3′	5.78, dt (14.0, 8.0)	131.4	6.27, dd (14.5, 10.0)	129.7	2.32, dt (7.0, 7.0)	33.6	1.45, m	30.4
4′	6.16, dd (14.0, 10.0)	132.2	5.89, dt (14.5, 7.5)	139.1	1.52, m	28.9	1.29, m	29.8
5′	6.08, dd (14.0, 10.0)	132.6	2.15, dt (7.5, 7.5)	35.0	1.35, m	31.6	1.19, m	32.4
6′	5.56, dq (14.0, 7.0)	127.9	1.47, m	22.4	1.35, m	22.6	1.21, m	23.2
7′	1.63, d (7.0)	18.4	0.94, t (7.5)	13.9	0.92, t (7.5)	14.2	0.83, t (7.0)	14.6
1″	3.48, d (7.5)	27.9	3.32, d (7.5)	27.4	3.32, d (7.5)	27.4	3.50, d (7.5)	27.8
2″	5.40, tm (7.5)	122.7	5.30, dm (7.5)	121.1	5.29, tm (7.5)	121.1	5.42, tm (7.5)	122.8
3″		133.7		134.2		134.1		133.6
4″	1.64, s	26.1	1.76, s	25.9	1.76, s	26.0	1.64, s	26.1
5″	1.59, s	18.0	1.70, s	17.9	1.70, s	17.9	1.60, s	18.0
2-OH	12.45, s		11.78, s		13.38, s		12.46, s	

## Data Availability

The data are available from the corresponding author.

## Supplementary Materials

The following supporting information can be downloaded at: https://www.mdpi.com/article/10.3390/foods12010049/s1, Figures S1–S12: HRESIMS, ^1^H and ^13^C NMR for compounds **1**–**4.**
