# Supplementary material for: Characterization of Hypolipidemic Phenol Analogues from Fermented Tea by Eurotium cristatum
Source: Foods. 2022 Dec 22;12(1):49. doi: 10.3390/foods12010049 (PMC9818934; doi:10.3390/foods12010049)
Supplement: Supplementary file 1 [file foods-12-00049-s001.zip › foods-2070095-supplementary.pdf]

SUPPLEMENTARY MATERIAL

Characterazation of Hypolipidemic phenol analogues from Fermented Tea by *Eurotium cristatum*

Fuhang Song [1,*], Wei Dai [1], Honghua Li [1], Xinwan Zhang [2], Xiuli Xu [2,*], Linlin Ma [3] and Long Wang [4]

Table of Contents

Figure S1. HRESIMS spectrum for **1** 3

Figure S2. ^{1}H NMR spectrum (500 MHz, Pyridine-d_5) of **1** 3

Figure S3. ^{13}C NMR spectrum (125 MHz, Pyridine-d_5) of **1** 4

Figure S4. HRESIMS spectrum for **2** 4

Figure S5. ^{1}H NMR spectrum (500 MHz, $CDCl_3$) of **2** 5

Figure S6. ^{13}C NMR spectrum (125 MHz, $CDCl_3$) of **2** 5

Figure S7. HRESIMS spectrum for **3** 6

Figure S8. ^{1}H NMR spectrum (500 MHz, $CDCl_3$) of **3** 6

Figure S9. ^{13}C NMR spectrum (125 MHz, $CDCl_3$) of **3** 7

Figure S10. HRESIMS spectrum for **4** 7

Figure S11. ^{1}H NMR spectrum (500 MHz, Pyridine-d_5) of **4** 8

Figure S12. ^{13}C NMR spectrum (125 MHz, Pyridine-d_5) of **4** 8

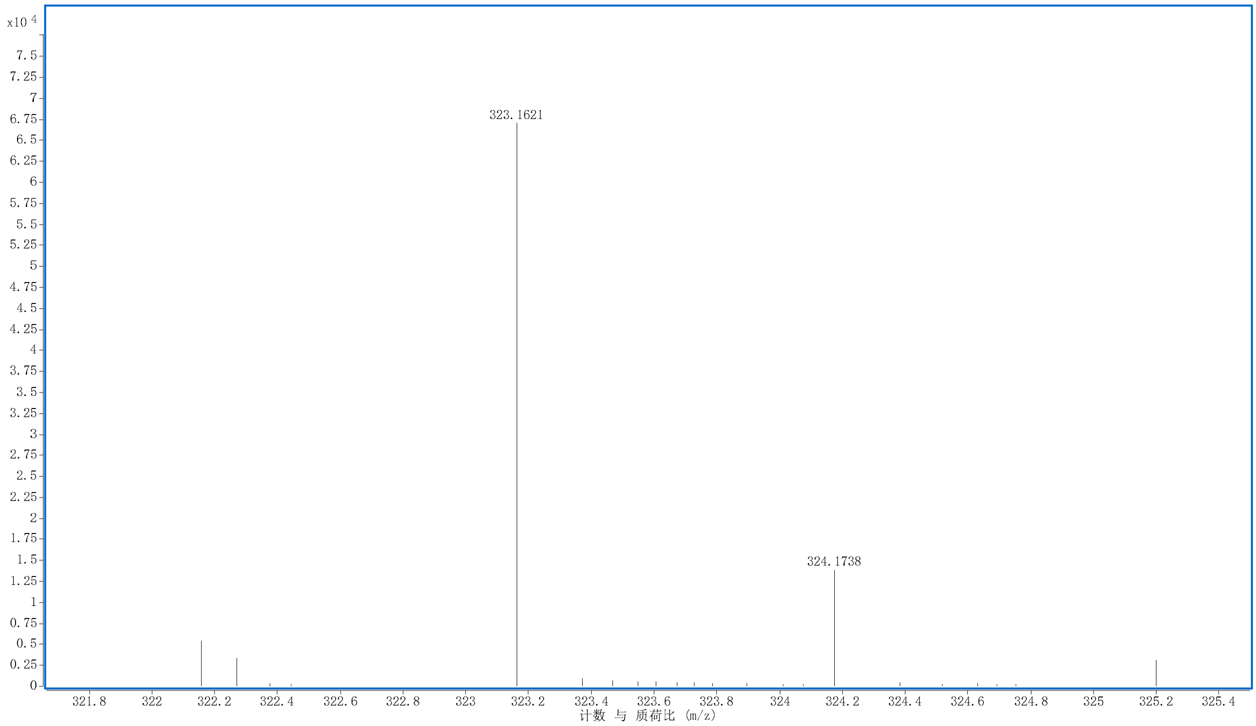

Figure S1. HRESIMS spectrum for **1**

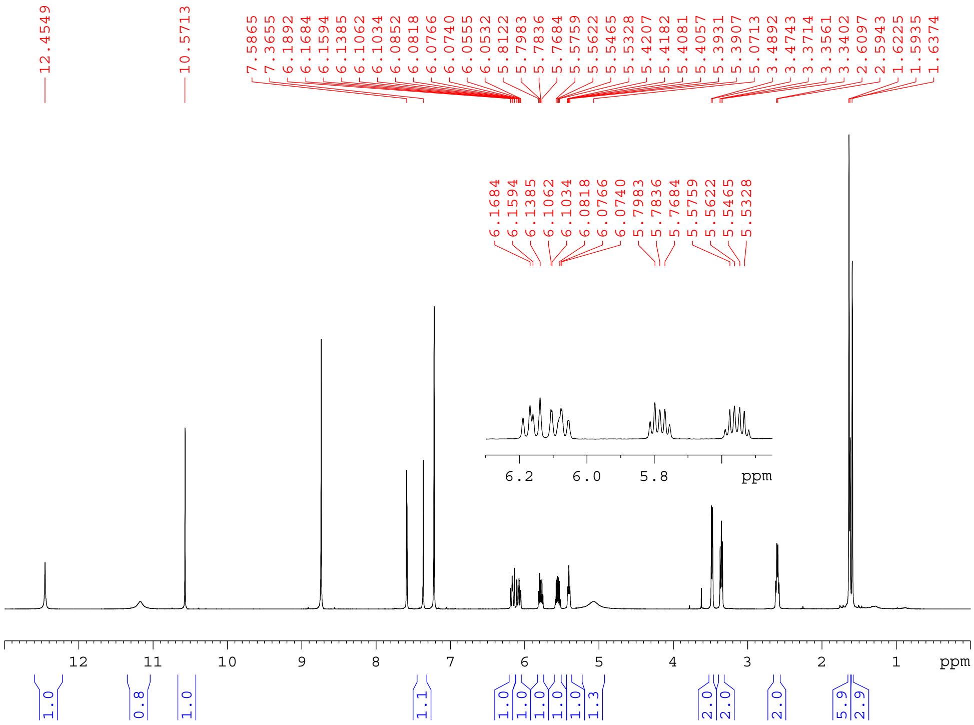

Figure S2. ^{1}H NMR spectrum (500 MHz, Pyridine-d_5) of **1**

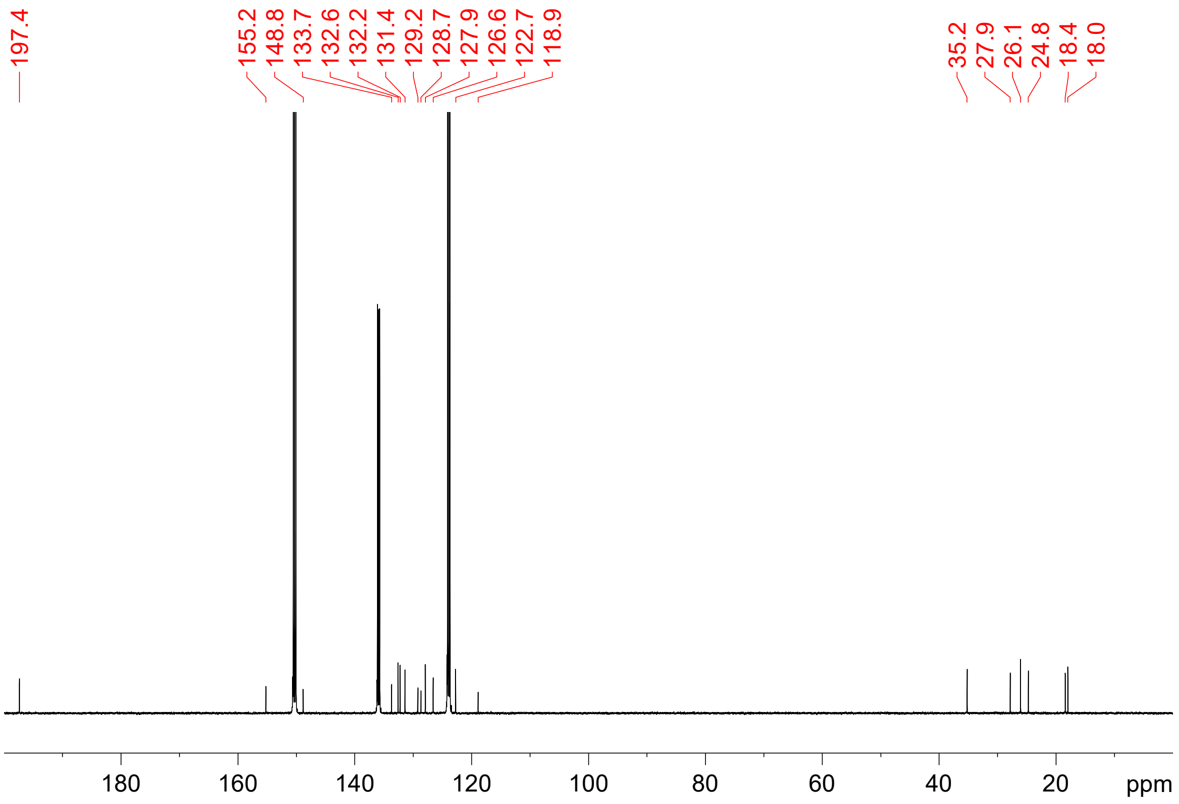

Figure S3. ^{13}C NMR spectrum (125 MHz, Pyridine-d_5) of **1**

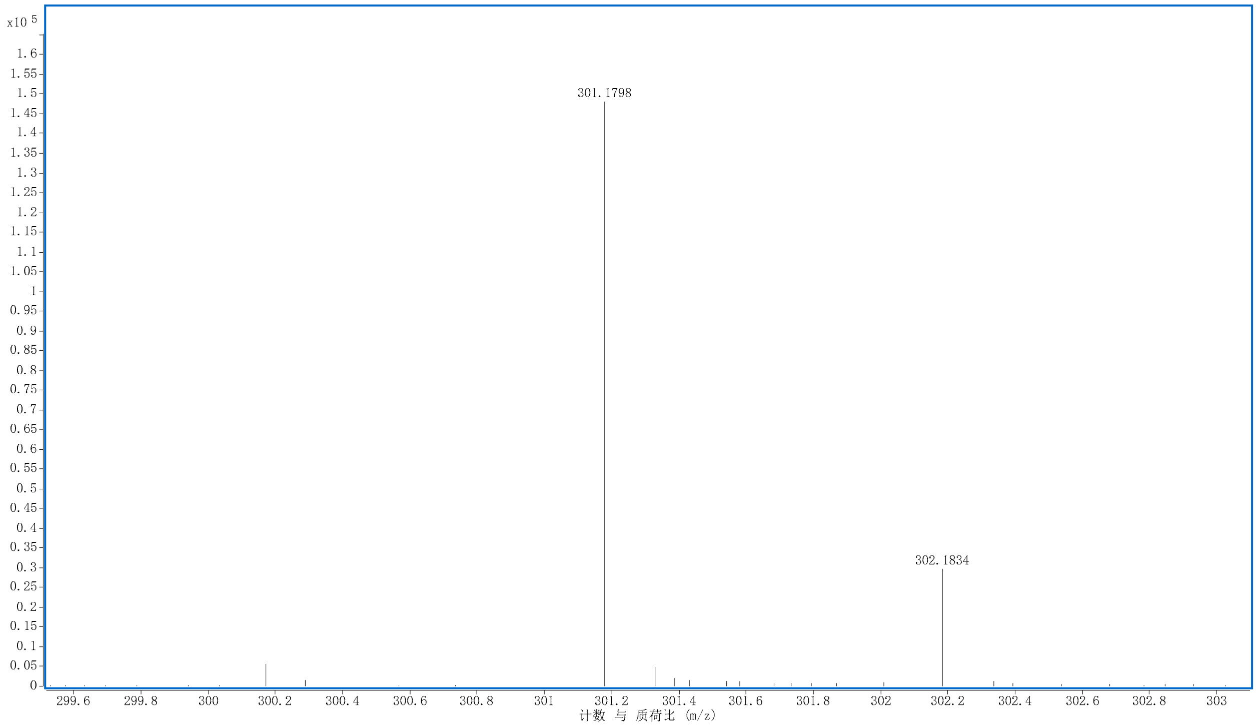

Figure S4. HRESIMS spectrum for **2**

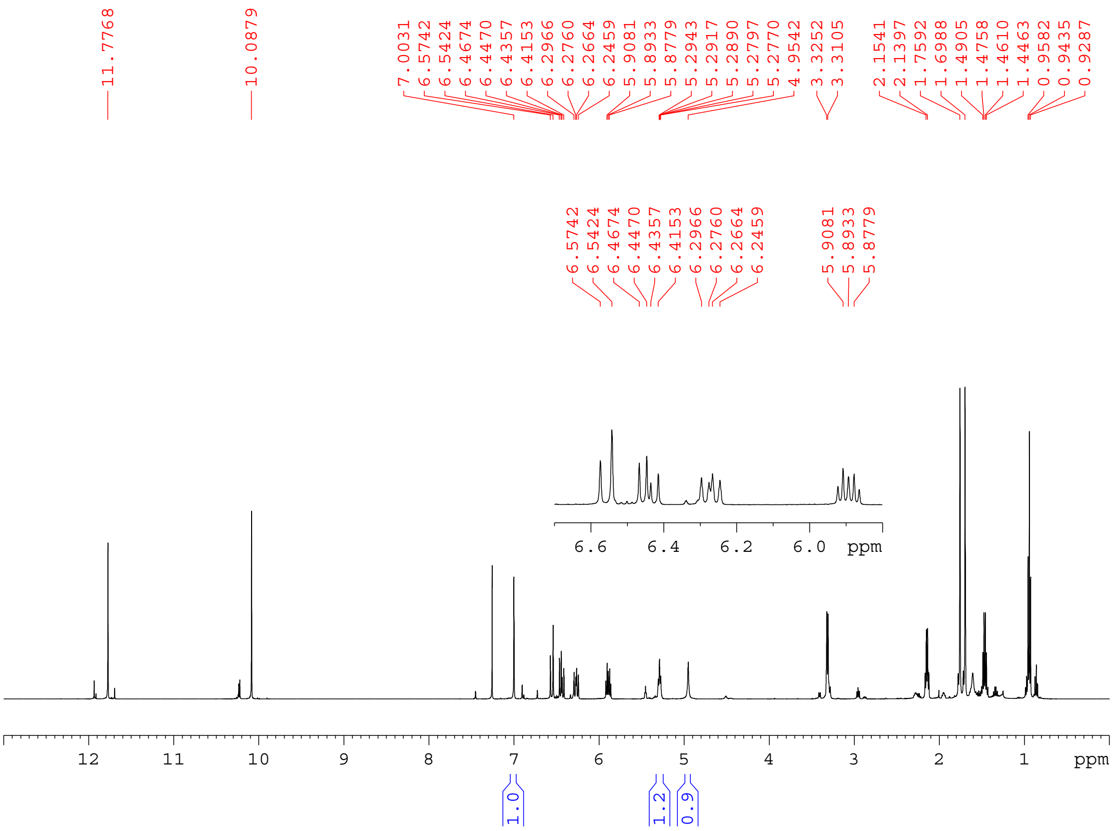

Figure S5. ^{1}H NMR spectrum (500 MHz, $CDCl_3$) of **2**

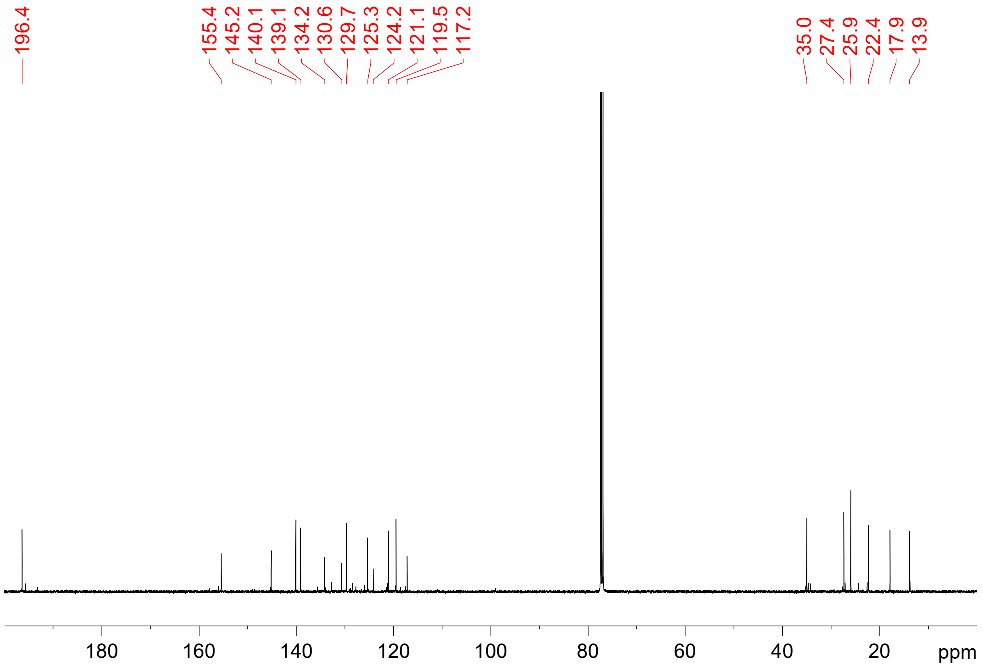

Figure S6. ^{13}C NMR spectrum (125 MHz, $CDCl_3$) of **2**

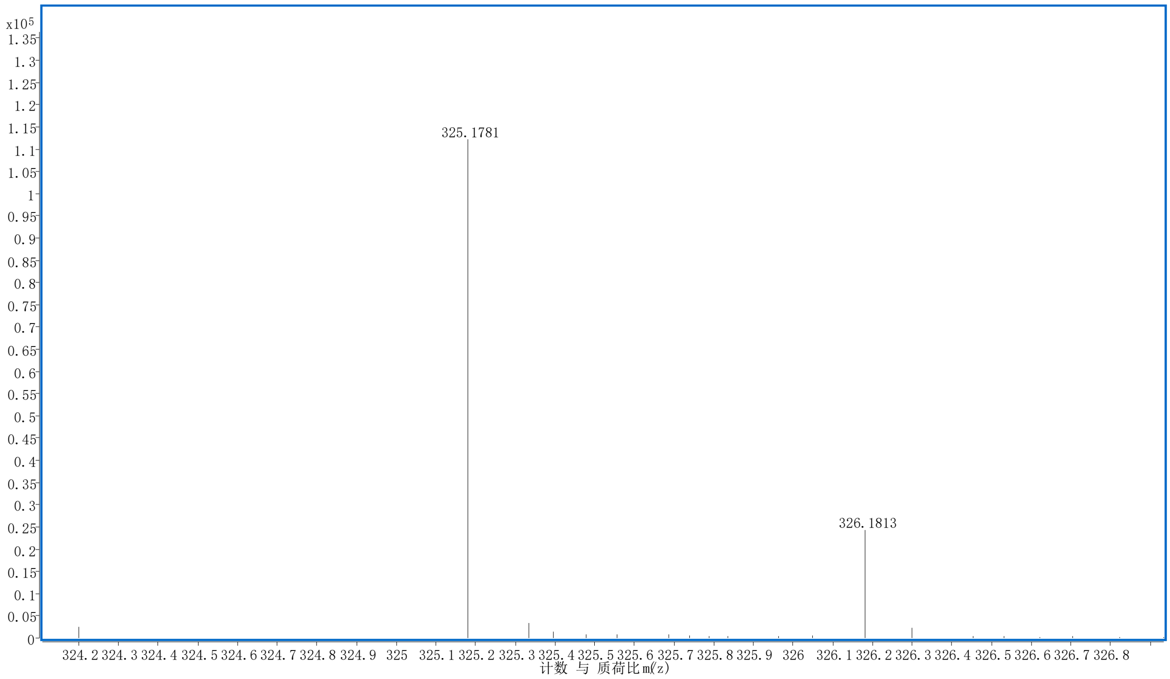

Figure S7. HRESIMS spectrum for **3**

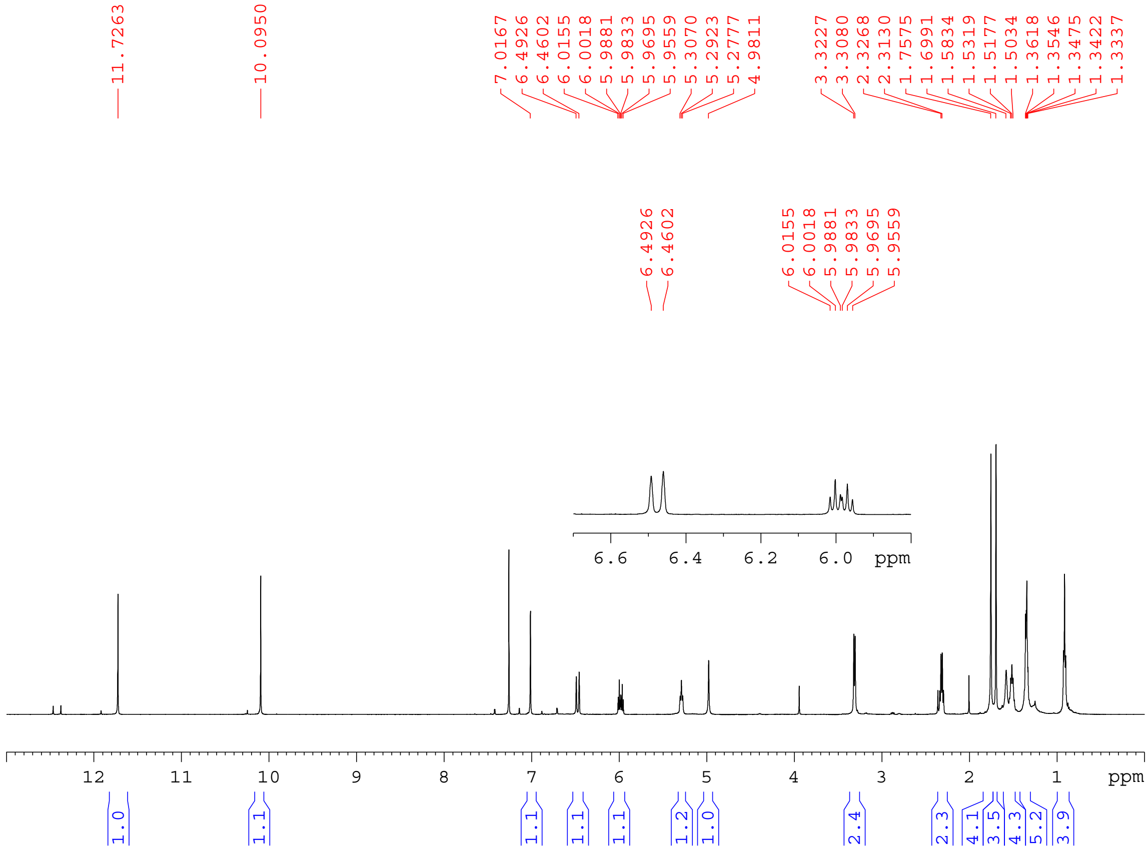

Figure S8. ^{1}H NMR spectrum (500 MHz, $CDCl_3$) of **3**

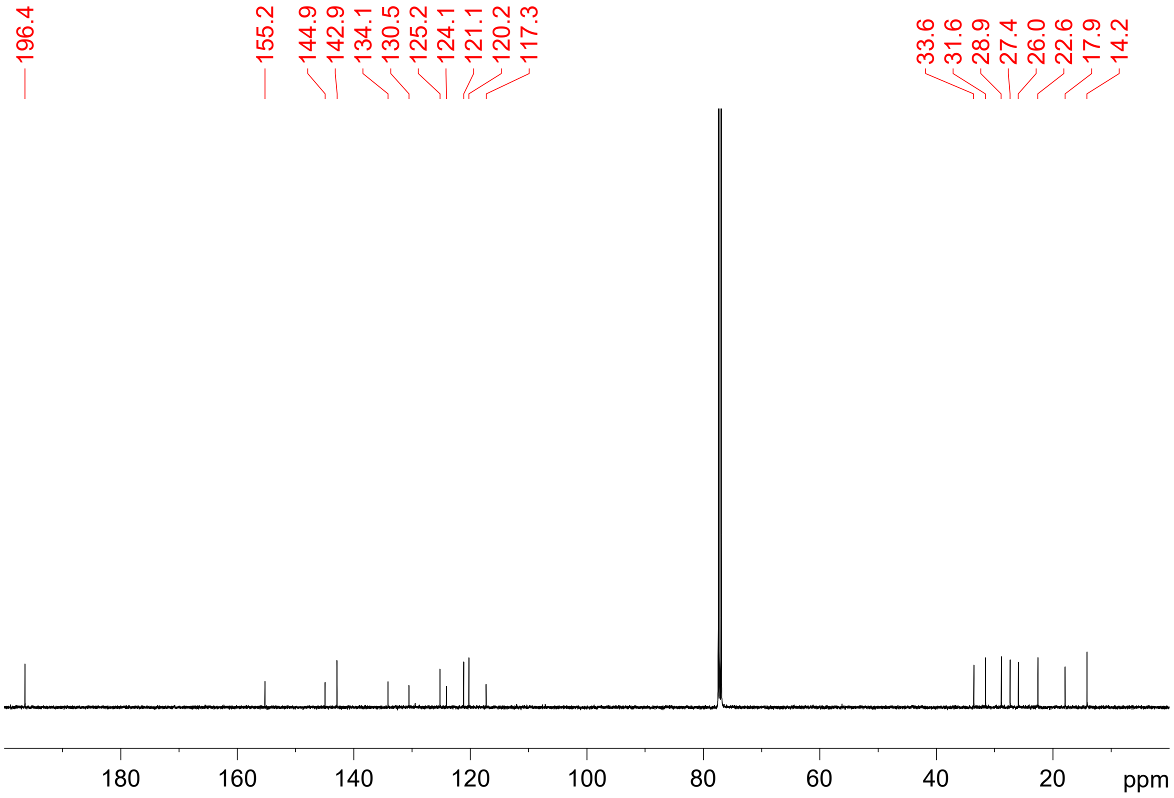

Figure S9. ^{13}C NMR spectrum (125 MHz, $CDCl_3$) of **3**

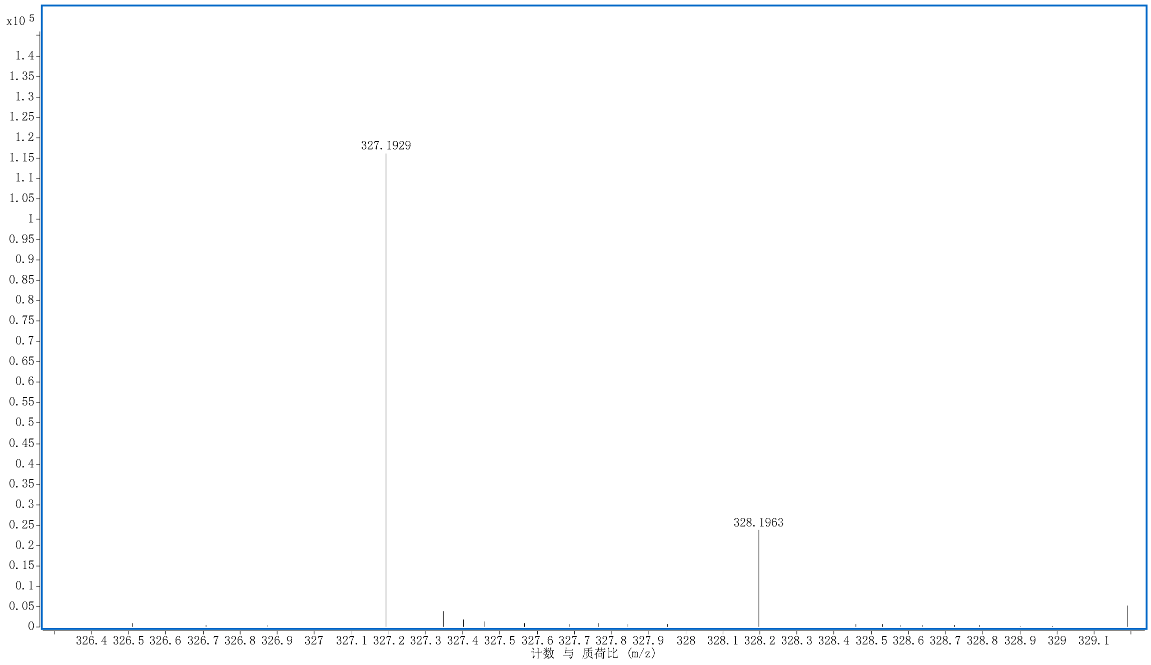

Figure S10. HRESIMS spectrum for **4**

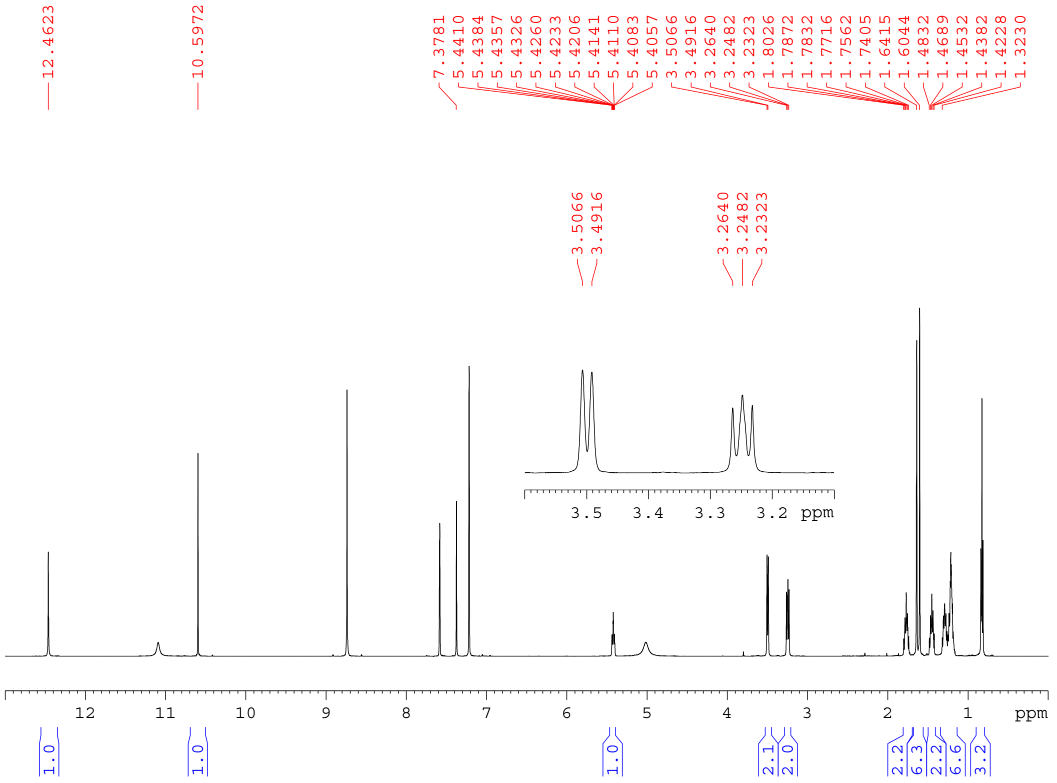

Figure S11. ^{1}H NMR spectrum (500 MHz, Pyridine-d_5) of **4**

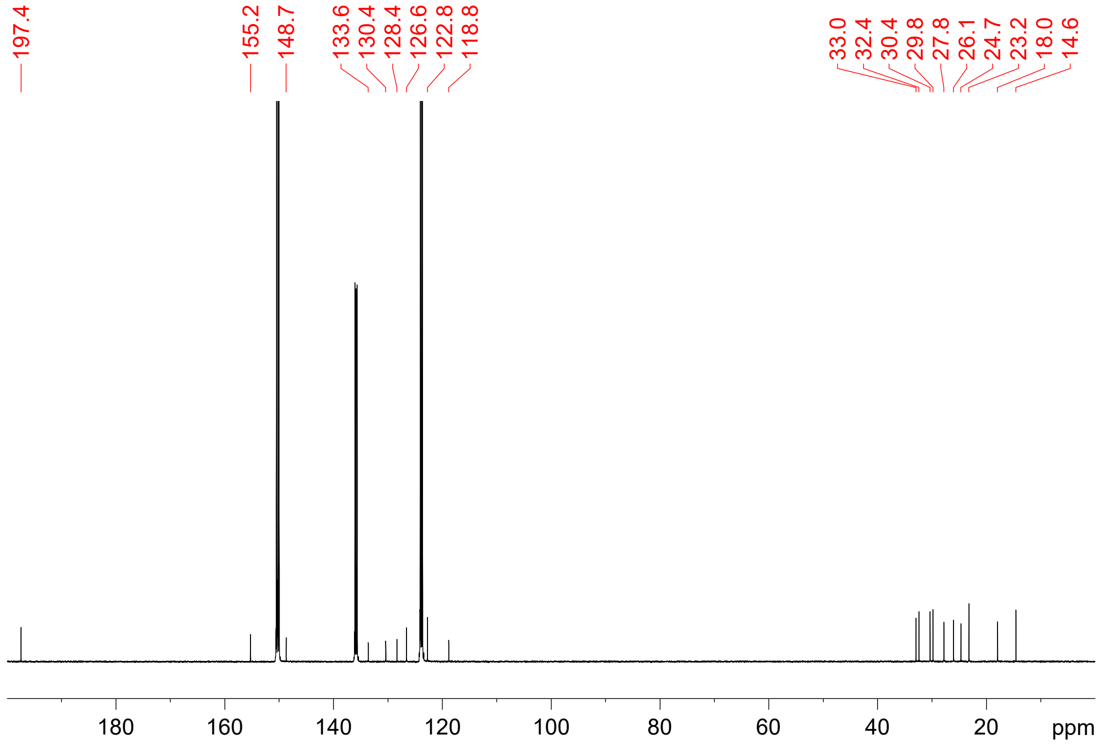

Figure S12. ^{13}C NMR spectrum (125 MHz, Pyridine-d_5) of **4**
